# The Structure of 2,6-Di-*tert*-butylphenol–Argon by Rotational Spectroscopy

**DOI:** 10.3390/molecules28248111

**Published:** 2023-12-15

**Authors:** Wenqin Li, Assimo Maris, Sonia Melandri, Alberto Lesarri, Luca Evangelisti

**Affiliations:** 1 Departamento de Química Física y Química Inorgánica, Facultad de Ciencias—I.U. CINQUIMA, Universidad de Valladolid, Paseo de Belén 7, 47011 Valladolid, Spain; wenqin.li@uva.es; 2 Department of Chemistry “G. Ciamician”, University of Bologna, Via F. Selmi 2, 40126 Bologna, Italy; assimo.maris@unibo.it (A.M.); sonia.melandri@unibo.it (S.M.); 3 Department of Chemistry “G. Ciamician”, University of Bologna, Via S. Alberto 163, 48123 Ravenna, Italy

**Keywords:** antioxidant, rotational spectroscopy, supersonic jet, non-covalent interactions

## Abstract

The molecular structure of a van der Waals-bonded complex involving 2,6-di-tert-butylphenol and a single argon atom has been determined through rotational spectroscopy. The experimentally derived structural parameters were compared to the outcomes of quantum chemical calculations that can accurately account for dispersive interactions in the cluster. The findings revealed a π-bound configuration for the complex, with the argon atom engaging the aromatic ring. The microwave spectrum reveals both fine and hyperfine tunneling components. The main spectral doubling is evident as two distinct clusters of lines, with an approximate separation of 179 MHz, attributed to the torsional motion associated with the hydroxyl group. Additionally, each component of this doublet further splits into three components, each with separations measuring less than 1 MHz. Investigation into intramolecular dynamics using a one-dimensional flexible model suggests that the main tunneling phenomenon originates from equivalent positions of the hydroxyl group. A double-minimum potential function with a barrier of 1000 (100) cm^−1^ effectively describes this extensive amplitude motion. However, the three-fold fine structure, potentially linked to internal motions within the tert-butyl group, requires additional scrutiny for a comprehensive understanding.

## 1. Introduction

Materials undergo continuous exposure to oxidative stress induced by sunlight, atmospheric oxygen, or adverse environmental conditions. This oxidative stress initiates the generation of reactive oxygen species (referred to as ROSs), including oxygen radicals such as hydroxyl (HO·), superoxide anion (O_2_^−^), alkoxyl radicals (RO·), and peroxyl radicals (ROO·). Additionally, there are non-radical oxidants that can easily transform into radicals [1], such as hydrogen peroxide (H_2_O_2_), singlet oxygen (O_2_^−^), hypochlorous acid (HOCl), or peroxynitrite (ONOO^−^). Consequently, these ROSs can bring about various alterations in the living tissues or non-living materials they come into contact with. Antioxidants are a family of molecular compounds that have the property of inhibiting oxidative processes by neutralizing free radicals and thus protecting materials [2]. These compounds are therefore ubiquitous in nature, and generally, even a minimal concentration is sufficient to keep the properties of the system unchanged. Several naturally occurring antioxidants belong to the family of phenolic compounds (PCs). These are, for example, an important source of nutrients for animals and can be found in fruits as well as in vegetables and serve to protect the molecules of living tissues such as lipids, proteins, and DNA. At the same time, they are used by humans as additives to preserve food or for other industrial purposes [3]. In fact, PCs are able to terminate the chain reactions of the production of free radicals by donating the hydrogen belonging to the hydroxyl group. Chemically, these PCs are divided into four main families characterized by different structural properties: flavonoids, stilbenes, lignans, and phenolic acids.

The awareness of the essential role performed by non-covalent interactions (NCIs) in these phenomena has grown within the scientific community over time. In fact, contemporary chemistry is currently deeply concentrated on comprehending these types of interactions. A chemical interaction is guided by a distinct molecular shape and charge distribution, and these attributes can be ascertained in an isolated gaseous state through rotational spectroscopy, supplemented by quantum chemical calculations [4,5].

In this work, we report the rotational spectrum of the weakly bound complex between the antioxidant 2,6-di-tert-butylphenol (henceforth 26BP) with Argon, recorded using the chirped-pulse-Fourier transform microwave (CP-FTMW) spectrometer and examined with the assistance of quantum mechanical calculations.

The main structural skeleton of 26BP is a phenol-like one [6].

A hydroxyl group is directly linked to a benzene ring. Located in positions 2 and 6 with respect to the hydroxyl, two tert-butyl groups are attached to the benzene ring. Similar to phenol and related molecules [7,8,9,10], the rotational spectrum analysis shows transitions that are split into distinct groups, separated by approximately 190 MHz. This splitting is primarily attributed to the tunneling motion associated with the hydroxyl group moving between two equivalent positions. From both physical and chemical perspectives, 26BP presents a significant opportunity to explore various types of non-covalent interactions (NCIs). This is due to its distinct sites that could potentially engage in such interactions, primarily the hydroxyl group and the aromatic electron π cloud. By selecting argon as the ligand, we delve into the inherent capability of 26BP to coordinate via van der Waals interactions. In this work, we report the rotational spectrum of the 26BP···Ar cluster. In general, complexes involving aromatic molecules with five- to six-membered rings tend to exhibit a certain degree of rigidity [11]. Rare gases typically do not disrupt their internal dynamics or structural characteristics. In order to understand how hydroxyl behaves as its chemical environment varies, we applied rotational spectroscopy to study its structural and electronic features.

## 2. Results

**Figure 1:**
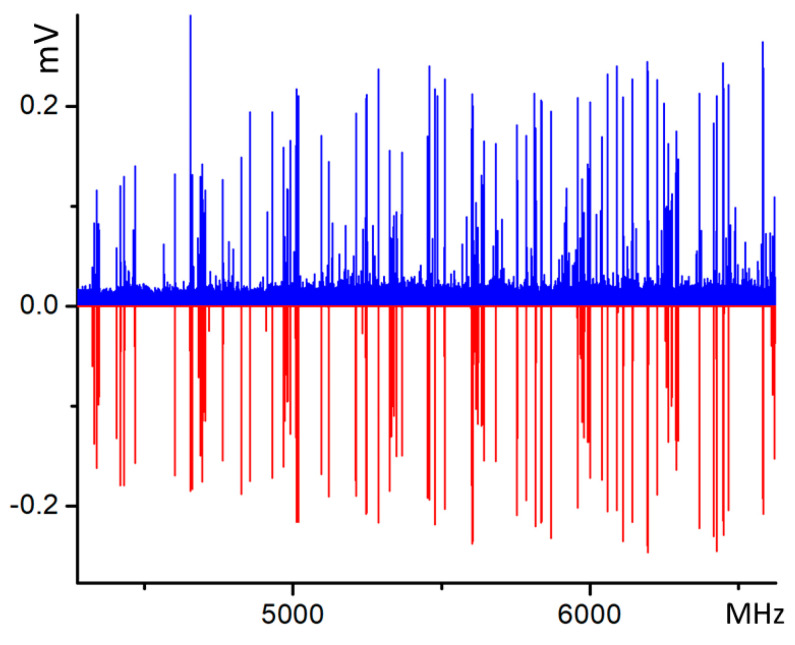
A portion of the broadband rotational spectrum of the 26BP–Ar complex. The blue upper traces correspond to the experimental spectrum (average of 1.5M FIDs). The lower red traces represent the simulated normal species produced with the experimental spectroscopic parameters at a rotational temperature of 0.8 K and the theoretical dipole moment components reported in Table 1.

**Figure 2:**
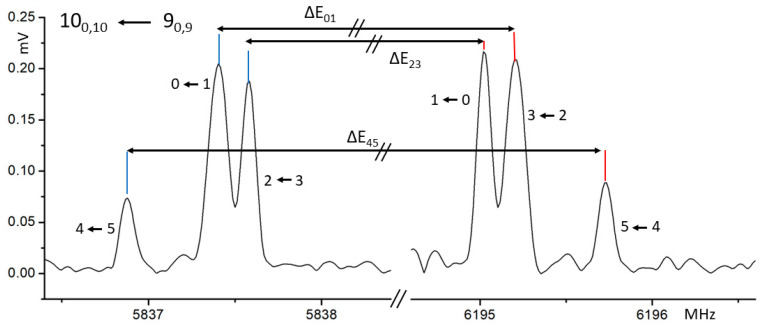
The characteristic shape of a transition of the 26BP–Ar complex. The main separation of components is noted at approximately 179 MHz, while the hyperfine structure appears much more compact.

The rotational spectrum of the molecular complex is shown in Figure 1 and was analyzed considering that the theoretical calculations (reported in Table 1) showed the dipole moment component *µ*_a_ to be the most intense for conformer 1, which is predicted to be the most stable one. Therefore, considering the spectrum prediction, a series of transitions split into various components was found. The main splitting of about 179 MHz separates the components into two groups. This splitting originates from the tunneling motion due to the hydroxyl group. This type of motion is similar to that observed in the monomer and molecules with the same structural framework, such as phenol [7,8,9,10] or *p*-cresol [12]. In the case of 26BP [6], however, each individual transition is further split into three components whose characteristics are described later in this paper. These splittings led to the assignment of three pairs of torsional states, denoted 0–1, 2–3, and 4–5 as can be seen in Figure 2. 

Besides the *R*-branch lines of *µ*_a_-type transitions, neither *µ*_b_-type or *µ*_c_-type transitions have been observed due to the weak predicted dipole moment components. 

All transitions were fitted using Watson’s semirigid-rotor Hamiltonian in the *I*^r^ representation and *S* reduction [13] with semi-rigid rotor terms (*H*^R^) and where each pair of torsional states was fitted using a two-state torsion-rotation coupled Hamiltonian, which led to a specific torsional energy difference Δ*E*_ij_ (where i and j indicate the torsional states involved), and Coriolis coupling term *F*_ab_ determined in the reduced-axis system of Pickett [14]:

(1) $$H=H_{ij}^{R}+H_{ij}^{int}\;$$

where:

(2) $$H_{ij}^{int}=∆E_{ij}+F_{ab,ij}\left(P_{a}P_{b}+P_{b}P_{a}\right)\;\mathrm{with}\;ij\;=\;01,\;23,\;45$$

and *P*_α_ (with α can be *a*, *b*, or *c*) are the angular momentum operators. All the resulting spectroscopic parameters are reported in Table 2 while the list of all rotational transitions with the relative quantum numbers and the residual error is reported in the Supplementary Materials. 

## 3. Discussion

Based on computational calculations, the conformers of the 26BP–Ar complex can be categorized into two distinct types, depending on the position of the argon atom with respect to the plane of symmetry of the benzene ring. In conformers I and V, unlike the others, the argon atom is situated off the plane of symmetry of the benzene ring. In the first conformer, the argon atom is positioned above the benzene ring, engaging with the electron cloud. This arrangement bears resemblance to observations made in adducts involving aromatic rings like benzene or pyridine with noble gases. Conversely, conformer V features the argon atom positioned at a distance from the symmetry plane, interacting with the *tert*-butyl group. In conformers II, III, and IV, the argon atom resides within the symmetry plane of the benzene ring. Notably, in this scenario, the movement of the argon atom around the molecule is of interest, primarily occurring within the portion where the hydroxyl group is oriented.

It is evident from the experimental rotational constants that the best match is found with those of conformer I, which is also the lowest in energy. Additionally, given that the observed spectrum corresponds to *µ*_a_-type, identifying the observed conformer becomes straightforward.

Rotational spectroscopy is inherently a high-resolution technique and the small mass variation in the mass of the molecular system is detectable as it provides a different rotational spectrum. Over the years, several methods have been devised for determining the positions of atoms within a molecule. One widely employed approach involves applying Kraitchman’s equations [15], facilitating the derivation of the *r*_s_ structure. The primary merit of this method lies in its lack of a priori assumptions, with the obtained values being reliably reproducible based on experimental data [16]. While the signs of atomic coordinates remain indeterminate, they are typically easily inferred [17] via comparison to the computational calculations. Using this technique, the position of the argon atom can be derived from the set of rotational constants of the monomer. In this case, it would involve the fictitious replacement of a dummy atom of mass zero with an argon atom of mass number 40. The results obtained provide the coordinates of the argon atom in the principal system of inertia (PAS) of the 26BP monomer. By approximating that the molecular structure of the monomer 26BP is not modified by complexation with the argon atom, it is possible to rototranslate the system calculated with Kraitchman’s coordinates in order to superimpose it with the previously calculated 26BP–Ar complex. This provides the coordinates of the Argon atom calculated by Kraitchman’s method in the PAS of the molecular complex. The resulting parameters of Kraitchman’s equations are compared in Table 3 with the equilibrium structure.

The good matching of the results provides a direct indication of the position of the Ar atom above the ring structure of 26BP.

**Figure 3:**
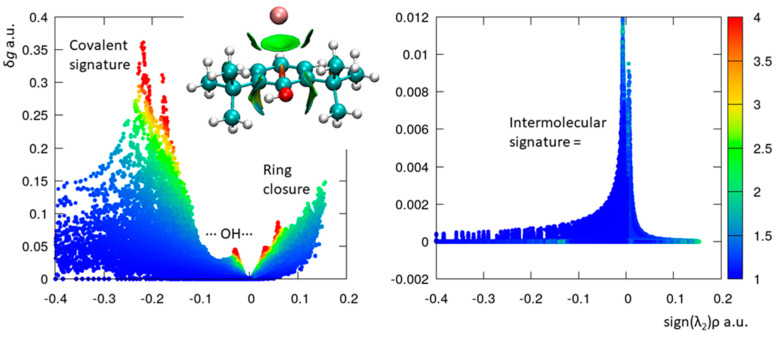
The 26BP–Argon complex optimized at the B3LYP-D3(BJ)/def2-TZVP level of theory, with δ*g^intra^* = *f*(sign(λ_2_)ρ) shown on the left and δ*g^inter^* = *f*(sign(λ_2_)ρ) shown on the right. The color coding is based on *qg* in the following range: 1 < *qg* < 4.

In contemporary times, rotational spectroscopy leverages the advancements and achievements of computational chemistry to accurately interpret outcomes. Simultaneously, spectroscopic data serve as a yardstick for evaluating quantum chemical theories, resulting in a productive synergy. In this current study, the modeling of 26BP and its complex with argon occurs at the B3LYP-D3(BJ)/def2-TZVP levels of computation. Given the substantial agreement between the theoretical parameters and the experimental ones, especially when considering the significant effects of large amplitude motion and assuming that the selected methods aptly depict our systems, there is potential for further theoretical analyses to be conducted, aiming to extract insights into the nature of non-covalent interactions. In this case, IGMPlot was applied [18]. IGMPlot utilizes the independent gradient model (IGM) and its associated local descriptor δ*g*. The IGM methodology examines the gradient of the electron density (ED) within a molecular system, aiming to pinpoint spatial areas where chemical interactions occur. The IGM δg descriptor quantifies the clash in ED between two designated sources of fragments (atoms or molecules). This descriptor captures the tendency of electrons to be mutually shared by both interacting entities. In practice, when plotting δg against the signed ED in a two-dimensional representation (as depicted in Figure 3), distinct peaks (δ*g* peak) emerge, forming a unique pattern that signifies the interactions within the system. The values of δ*g* peak can be linked to specific interaction types, ranging from non-covalent to covalent, on an absolute scale. Moreover, the integrated Δ*g* value provides an assessment of the strength of the interaction (for more details, refer to ref. [18]). For clarity, the two interaction plots have been separated. In Figure 3, the graph on the left shows interactions that solely pertain to 26BP. Notably, interactions resulting from covalent bonds among the molecule’s atoms are observable at values above −0.1 a.u., while NCIs between the hydroxyl group and the two *tert*-butyl groups are noticeable at lower values. These interactions and their values appear similar between the monomeric 26BP and the 26BP complexed with the argon atom. This is consistent with the observation that the presence of the argon atom does not significantly perturb the structure of 26BP. Conversely, in the graph on the right, the NCI between the argon atom and 26BP is evident. It is important to note the varying peak height scales between these two plots: approximately 0.4 atomic units for C-C bonding (left, δ*g*-intra) and 0.012 atomic units for van der Waals interaction (right, δ*g*-inter).

Supermolecular methods offer a means of quantifying the energy associated with NCIs. The most basic approach is the subtractive method, where the intermolecular binding energy (*D*_e_) is calculated by determining the disparity between the energy of the binary molecular complex (A–B) and the energy of the individual constituent units (A and B) in their most stable arrangement:

*D*_e_ = (*E*_A−B_) − (*E*_A_ + *E*_B_) (3)

Similarly, the interaction energy is computed as the difference between the energy of the molecular complex and the energy of the isolated monomers in the geometry of the complex (A* and B*):

*E*_int_ = (*E*_A−B_) − (*E*_A_^∗^ + *E*_B_^∗^) (4)

The results are reported in Table 3. For this complex, *E*_int_ and *D*_e_ hold almost the same value. This further substantiates that no structural relaxation occurs upon complexation in the argon complex.

A distinct perspective is presented by the Symmetry-Adapted Perturbation Theory (SAPT), which regards the total interaction energy as a perturbation to the overall system energy [19]. An advantage of this approach over the subtractive one lies in its ability to naturally eliminate the basis set superposition error from the interaction energy. We employed an advanced SAPT methodology (SAPT2 + 3/aug-cc-pVDZ-RI) integrated into the PSI4 package [20] to determine the *E*_int_ values detailed in Table 4, inclusive of their electrostatic, exchange-repulsion, induction, and dispersion constituents. When examining the argon complex, the calculated interaction energies using SAPT reveal that the main stabilizing factor arises from dispersion effects. Its total value of 7.94 kJ/mol is well within the range of weak NCIs.

The rotational spectrum of the molecular complex is dominated by a tunneling phenomenon due to the equivalent positions of the hydrogen atom of the hydroxyl group with a spacing of about 179 MHz. This tunneling phenomenon is similar to that observed for phenol, whose splitting is, however, around 60 MHz. Over the years, various theories have been considered to explain the phenol phenomenon, including the motion of the hydroxyl above and below the plane of the ring. Currently, we agree with a motion attributed to the two equivalent positions of the hydroxyl in the plane of the ring. In this approximation, the experimental data are well reproduced considering a double minimum potential function with a V_2_ ~ 1200 cm^−1^.

With regard to the 26BP monomer, the observation of interstate transitions in the *µ*_a_ component, and only intrastate transitions of the *µ*_b_ component agrees with the fact that the motion is due to the internal rotation of the hydroxyl group, whose potential can be described by a function with two equivalent symmetric minima in which the hydroxyl hydrogen is coplanar with the benzene ring. This motion is also corroborated by the number of ^13^C isotopologues observed (12 out of 14 possible isotopologues), by their relative intensities with varying positions (two couples of ^13^C have double intensities compared to the other isotopologues), and by the maintenance of this splitting for the positions that do not alter the molecular symmetry with respect to the parent species. During the transition from the 26BP monomer to the complex with argon, the primary splitting remains, decreasing from 190 MHz to approximately 179 MHz. 

To simulate this internal motion and accurately replicate the experimental data, the decision was made to employ the monodimensional flexible model proposed by Meyer, which features a similar function used for the 26BP monomer [21]. In essence, the model posits that molecules undergoing significant amplitude motion can be described by a potential function governing these intramolecular dynamics. The internal motion of the hydroxyl group is captured by employing a twofold potential function:

*V*(τ) = ½ *V*_2_ · (1 − cos 2·*τ*) (5)

where *V*_2_ is the barrier between the two equivalent minima and *τ* is the torsional coordinate (C-C-O-H). Regarding the remaining structural parameters, they were constrained to their theoretical values (calculated at the B3LYP-D3(BJ)/def2-TZVP level) by enforcing a *C*_s_ arrangement for all atoms except the hydroxyl hydrogen atom. This model accurately replicates the observed splitting (179 cm^−1^) when the barrier is set at 889 cm^−1^.

**Figure 4:**
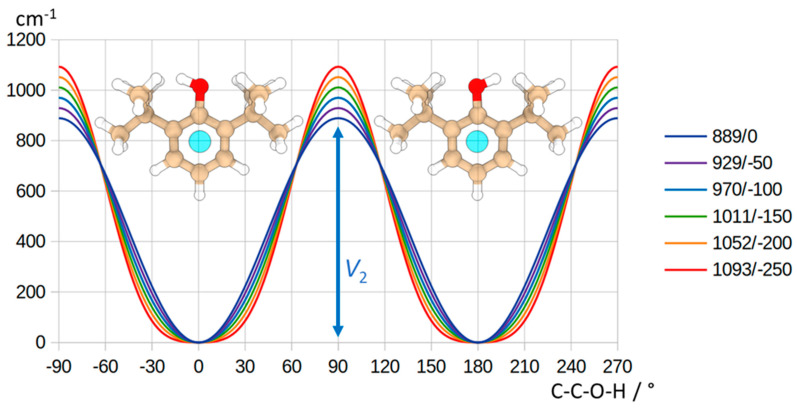
Schematic diagram of the double minimum potential function of the 26BP–Ar complex. Possible flexible model potential energy functions are proposed to replicate the observed splitting (with the corresponding *V*_2_ and *V*_4_ values provided in the legend (*V*_2_/*V*_4_ in cm^−1^)). The two conformations of the 26BP–Ar complex correspond to two equivalent potential minima.

Similar to the 26BP monomer, examining the theoretical double-minimum torsional potential function obtained through a dihedral angle *τ* scan of 10° at the B3LYP-D3(BJ)/def2-TZVP level of calculation (Figure 4) reveals that the minimum wells are slightly broader, while the barrier is narrower compared to the model described in the previous equation. This influence can be addressed by introducing a negative *V*_4_ term, outlined as follows:

*V*(*τ*) = ½ *V*_2_·[1 − cos(2·*τ*)] + ½ *V*_4_·[1 − cos(4·*τ*)] (6)

The potential barrier calculated at this computational level can be reproduced using the following equation with the values *V*_2_ = 992 cm^−1^ and *V*_4_ = −91 cm^−1^. However, due to the experimental energy splitting, fitting both parameters independently is not feasible. Instead, a range of parameter pairs can be identified that replicate the splitting. The depicted curves in Figure 4 demonstrate that, with increasing negativity in *V*_4_, the barrier height rises to uphold a consistent splitting value. As a result, the value derived from the basic model sets the minimum threshold for the hydroxyl internal rotation barrier (*V*_2_ = 889 cm^−1^), estimating it at approximately 1000(100) cm^−1^. It is worth emphasizing that incorporating structural relaxation within the model has the potential to modify this estimation.

It is intriguing to observe that the barrier values obtained differ by merely 1 cm^−1^ from the barrier values of the 26BP monomer. This observation highlights how the presence of the Argon atom, which forms an exceedingly weak non-covalent bond, has a limited impact on the internal motion connected with the hydroxyl group.

Moreover, similar to the 26BP monomer, the enigmatic hyperfine splitting persists in the complex. The division of these transitions into three components spaced by a few kilohertz still eludes complete understanding. As depicted in Figure 2, the splitting of these transitions maintains regularity across the entire measurement range, with the transition of lower intensity positioned at the extremes and the one with higher intensity in the central position of each triplet. The relative intensity ratio across all transitions averages around 0.7:1:0.2. Several attempts have been made to comprehend this phenomenon, including efforts to model the potential rotation of tert-butyl groups. with various available software, but this phenomenon is not yet understood [22,23]. It is likely that currently, this motion characteristic of 26BP alone cannot be modeled with the known Hamiltonians but will require an in-depth study and the advanced application of permutation–inversion group to interpret and model the possible large-amplitude motion coupled to the tunneling of the hydroxyl group.

## 4. Materials and Methods

For this analysis, a sample of 2,6-di-*tert*-butylphenol (CAS:128-39-2) was used, which is an odorless and colorless solid that was obtained from Merck. The declared purity was greater than 97% and considering the high resolution of the rotational technique, the sample did not require any further purification.

The molecular rotational resonance spectrum was recorded using the broadband (chirped-pulse) Fourier transform spectrometer at the University of Valladolid that has been previously described [24]. The sample was heated to 403 K and a mixture of argon and helium at 0.3 MPa was passed over the vapors produced in situ.

Before searching for the rotational lines in the spectrum, we conducted preliminary model calculations to establish plausible stable conformations. To achieve this, we first employed the distributed polarizability model (DPM) [25], followed by computational calculations in the proximity of the DPM minima.

(a) The DPM computations were executed utilizing the software RGDMIN [26]. The configuration of 26BP was held constant at the theoretical structure, whereas the distance (RCM) between its center of mass (CM) and the Ar noble gas was allowed to adjust freely in order to minimize energy across the complete spectrum of *θ* = 0–180°, *φ* = 0–360°, incrementing in steps of *θ* = *φ* = 10°. RCM, *θ*, and *φ* represent the spherical coordinates.
(b) Using Gaussian16 [27], the relative minima obtained from RGDMIN have been optimized in order to guide the experimental assignment. The results obtained with the DFT B3LYP method with the addition of the D3 method developed by Grimme et al. [28] and the Becke–Johnson damping function [29,30] have been reported in Table 1. The Weigend and Ahlrich’s def2-TZVP basis set [31] was used as previous work has highlighted the accuracy of the method for experimental purposes [32,33,34]. Table 1 reports the spectroscopic parameters obtained, including the rotational constants and the dipole moment components, which are necessary for the prediction of the rotational spectrum. The frequency calculation at B3LYP-D3(BJ)/def2-TZVP was performed with the harmonic approximation. All the theoretical structures are reported in the Supplementary Materials.

## 5. Conclusions

Our examination of the rotational spectrum of the 26BP–Argon complex has unveiled several instances of tunneling effects. These effects primarily stem from the torsional movement linked to the hydroxyl group. Each transition is additionally divided into three components, which can be understood as pairs of torsional states. Concerning the molecular structure, rotational spectroscopy has facilitated the determination of the molecular arrangement based on the rotational constants. By employing Kraitchman’s equations for analysis, we were able to validate that the observed conformer corresponds to the arrangement where the Argon atom is positioned above the plane of the ring, aligned with the hydroxyl group.

The intramolecular dynamics of 26BP–Argon include the phenomenon of tunneling due to the symmetric positions of the hydroxyl group. The potential function used to model internal rotation effectively captured the experimental data. It featured a double minimum potential with a barrier of approximately 1000(100) cm^−1^ between the equivalent minima. However, we were unable to explain the division of each transition into three components. This specific conformation, while altering the apparent symmetry of the system from *C*_2v_ (as seen in the isolated monomer) to *C*_s_ (due to the presence of Argon in the complex), does not exert any influence on the nature or the extent of the splitting observed in the spectrum for each individual transition. This behavior closely resembles what has been observed in isolated 26BP molecules, indicating that the presence of the argon atoms does not significantly affect the internal dynamics of the monomer. In the future, this information may be used to fully understand the origin of these splittings, which is currently unknown. To address this, additional exploration is necessary, involving the application of permutation–inversion group theory [35,36] to comprehend the significant amplitude motions linked to both the hydroxyl and *tert*-butyl group tunneling. 

## Figures and Tables

**Table 1:** Theoretical spectroscopic constants of the 26BP–Ar complex’s conformation. The figures reported at the bottom show the calculated structures.

	I	II	III	IV	V
*A*/MHz	576.17	489.09	649.65	938.74	832.23
*B*/MHz	345.94	334.57	231.54	182.21	185.64
*C*/MHz	298.78	218.11	184.85	163.80	179.99
*µ*_a_/D	−1.84	1.76	1.54	2.04	1.73
*µ*_b_/D	−0.14	0.75	−1.17	0.15	−0.29
*µ*_c_/D	0.23	0.00	0.00	0.00	0.58
Δ*E*/cm^−1^	0 *	315	443	460	486
Δ*E*_0_/cm^−1^	0 *	308	411	434	450
	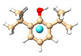	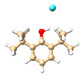	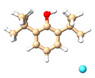	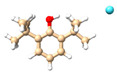	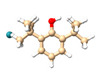

* The absolute values of the energies are −1149.830107 hartrees and −1149.500398 hartrees (ZPE-corrected relative energies).

**Table 2:** Experimental spectroscopic constants of the 26BP–Ar complex. The figure at the bottom shows the calculated structure of 26BP–Ar, and the blue arrow shows the total electric dipole moment of the conformer.

States	0,2,4	1,3,5
*A*/MHz	563.740(4) ^[a]^	
*B*/MHz	345.3613(3)	345.3597(4)
*C*/MHz	294.9384(3)	294.9382(3)
Δ*E*^01^/MHz	178.933(4)	
Δ*E*^23^/MHz	178.757(4)	
Δ*E*^45^/MHz	179.463(4)	
*F*_ab_^01^/MHz	1.168(4)	
*F*_ab_^23^/MHz	1.170(4)	
*F*_ab_^45^/MHz	1.162(4)	
*D*_J_/Hz	17(1)	
*D*_JK_/kHz	0.21(1)	
*σ* ^[b]^/kHz	9	
*N* ^[c]^	442	
*µ*_a_/*µ*_b_/*µ*_c_ ^[d]^/D	y/n/n	
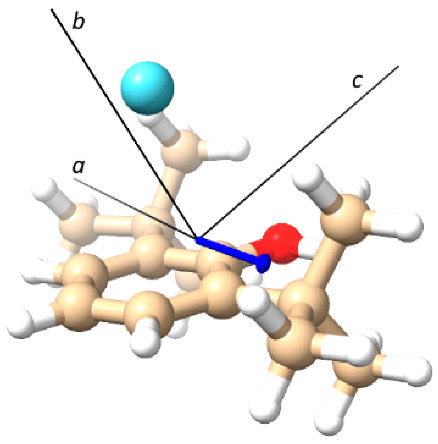		

^[a]^ Errors in parentheses are expressed in units of the last digits. ^[b]^ Standard deviation of the fit. ^[c]^ Number of fitted transitions. ^[d]^ *µ*_a_/*µ*_b_/*µ*_c_ are the electric dipole moment components along the principal inertial axes *a*, *b*, and *c*.

**Table 3:** Experimental substitution coordinates (*r*_s_) and theoretical equilibrium coordinates (*r*_e_, at B3LYP-D3(BJ)/def2-TZVP level of theory).

	*a* (Å)		*b* (Å)		*c* (Å)	
	|*r*_s_|	*r*_e_	|*r*_s_|	*r*_e_	|*r*_s_|	*r*_e_
Ar (PAS monomer)	0.57i	+0.029	0.854(1)	+0.782	3.496(1)	−3.444
Ar (PAS complex)	0	−0.042	2.950(1)	+2.883	0.554(1)	+0.661

**Table 4:** Theoretical binding and interaction energies are provided in kJ/mol at the B3LYP-D3(BJ)/def2-TZVP level of theory. Additionally, the exchange-repulsion, induction, and dispersion components obtained using the SAPT2 + 3/aug-cc-pVDZ-RI are reported.

	*D*_e_	*E*_int_	Electrostatic	Exch.–Repulsion	Induction	Dispersion	Total
26BP–Ar	−3.78	−3.80	−4.13	11.85	−1.03	−14.63	−7.94

## Data Availability

Data are contained within the article and Supplementary Materials.

## Supplementary Materials

The following supporting information can be downloaded at: https://www.mdpi.com/article/10.3390/molecules28248111/s1, Table S1: Measured rotational transitions of 2,6-di-tert-butylphenol–Ar complex. Table S2: Theoretical cartesian coordinates (Å) for conformer 1. Table S3: Theoretical cartesian coordinates (Å) for conformer 2. Table S4: Theoretical cartesian coordinates (Å) for conformer 3. Table S5: Theoretical cartesian coordinates (Å) for conformer 4. Table S6: Theoretical cartesian coordinates (Å) for conformer 5.
