# Supplementary material for: The Structure of 2,6-Di-tert-butylphenol–Argon by Rotational Spectroscopy
Source: Molecules. 2023 Dec 15;28(24):8111. doi: 10.3390/molecules28248111 (PMC10745844; doi:10.3390/molecules28248111)

Supplementary Materials

The Structure of 2,6-Di-*tert*-butylphenol–Argon by Rotational Spectroscopy

Wenqin Li [1], Assimo Maris [2], Sonia Melandri [2], Alberto Lesarri [1,*] and Luca Evangelisti [3,*]

[1] Departamento de Química Física y Química Inorgánica, Facultad de Ciencias—I.U. CINQUIMA, Universidad de Valladolid, Paseo de Belén 7, 47011 Valladolid, Spain; wenqin.li@uva.es
[2] Department of Chemistry "G. Ciamician", University of Bologna, Via F. Selmi 2, 40126 Bologna, Italy; assimo.maris@unibo.it (A.M.); sonia.melandri@unibo.it (S.M.)
[3] Department of Chemistry "G. Ciamician", University of Bologna, Via S. Alberto 163, 48123 Ravenna, Italy
* Correspondence: alberto.lesarri@uva.es (A.L.); luca.evangelisti6@unibo.it (L.E.)

Table S1. Measured rotational transitions of 2,6-di-tert-butylphenol – Ar complex, residuals according to fit of Table 1 in the main article (all values in MHz).

J'	K_{-1}'	K_{+1}'	v'	J''	K_{-1}''	K_{+1}''	v''	Frequency (MHz)	Residual (MHz)
7	1	7	0	6	1	6	1	4061.574	0.002
7	0	7	0	6	0	6	1	4073.646	0.004
7	1	7	1	6	1	6	0	4419.394	0.006
7	0	7	1	6	0	6	0	4431.478	0.010
8	0	8	0	7	0	7	1	4660.426	-0.004
8	1	8	0	7	1	7	1	4654.077	0.002
8	1	7	0	7	1	6	1	4930.574	0.002
9	1	9	0	8	1	8	1	5245.326	0.003
9	0	9	0	8	0	8	1	5248.515	0.001
6	1	5	1	5	1	4	0	4089.308	-0.003
6	2	4	1	5	2	3	0	4158.475	-0.004
7	2	6	0	6	2	5	1	4246.389	0.000
7	1	6	0	6	1	5	1	4338.861	-0.006
7	2	6	1	6	2	5	0	4602.518	0.005
7	1	6	1	6	1	5	0	4695.070	-0.005
6	3	3	1	5	3	2	0	4083.188	-0.001
7	3	5	0	6	3	4	1	4330.681	-0.005
7	5	3	0	6	5	2	1	4330.953	0.018
7	5	2	0	6	5	1	1	4331.184	0.009
7	4	4	0	6	4	3	1	4339.305	-0.004
7	4	3	0	6	4	2	1	4346.221	0.000
7	2	5	0	6	2	4	1	4467.722	-0.007
7	3	5	1	6	3	4	0	4688.096	0.000
7	5	3	1	6	5	2	0	4688.515	-0.020
7	5	2	1	6	5	1	0	4688.765	-0.011
7	4	4	1	6	4	3	0	4696.822	0.005
7	4	3	1	6	4	2	0	4703.735	0.000
7	3	4	1	6	3	3	0	4763.806	0.003
7	2	5	1	6	2	4	0	4825.226	-0.013
8	2	7	0	7	2	6	1	4854.827	0.000
8	3	6	0	7	3	5	1	4968.823	0.004
8	5	4	0	7	5	3	1	4981.601	-0.001
8	5	3	0	7	5	2	1	4982.538	-0.007
8	4	5	0	7	4	4	1	4991.027	-0.001
8	4	4	0	7	4	3	1	5009.082	0.000
8	1	8	1	7	1	7	0	5011.892	0.013
8	0	8	1	7	0	7	0	5018.253	0.010

8	3	5	0	7	3	4	1	5095.935	0.000
8	2	6	0	7	2	5	1	5120.182	-0.004
8	1	7	1	7	1	6	0	5287.357	-0.001
8	3	6	1	7	3	5	0	5326.050	0.006
8	6	3	1	7	6	2	0	5330.590	0.012
8	5	4	1	7	5	3	0	5339.048	0.005
8	5	3	1	7	5	2	0	5339.977	-0.009
8	4	4	1	7	4	3	0	5366.450	-0.002
8	3	5	1	7	3	4	0	5453.329	-0.004
9	2	8	0	8	2	7	1	5459.209	0.000
8	2	6	1	7	2	5	0	5477.548	-0.005
9	1	8	0	8	1	7	1	5510.615	0.000
9	3	7	0	8	3	6	1	5600.804	-0.003
9	0	9	1	8	0	8	0	5606.320	0.007
9	5	5	0	8	5	4	1	5634.447	-0.008
9	5	4	0	8	5	3	1	5637.428	-0.004
9	4	6	0	8	4	5	1	5642.383	0.000
9	4	5	0	8	4	4	1	5682.340	-0.004
9	2	7	0	8	2	6	1	5754.598	0.003
9	3	6	0	8	3	5	1	5785.787	0.005
9	2	8	1	8	2	7	0	5817.323	0.012
10	0	10	0	9	0	9	1	5837.404	-0.007
9	1	8	1	8	1	7	0	5868.786	0.005
9	6	4	1	8	6	3	0	5980.186	-0.018
9	6	3	1	8	6	2	0	5980.325	0.012
9	5	5	1	8	5	4	0	5991.717	-0.001
9	5	4	1	8	5	3	0	5994.706	0.006
9	4	6	1	8	4	5	0	5999.552	0.000
9	4	5	1	8	4	4	0	6039.568	-0.003
10	2	9	0	9	2	8	1	6058.521	0.000
10	1	9	0	9	1	8	1	6090.809	0.000
9	2	7	1	8	2	6	0	6111.745	-0.008
9	3	6	1	8	3	5	0	6143.067	-0.006
10	1	10	1	9	1	9	0	6193.650	-0.001
10	0	10	1	9	0	9	0	6195.206	0.005
10	3	8	0	9	3	7	1	6225.751	0.007
10	5	6	0	9	5	5	1	6289.240	0.002
10	4	7	0	9	4	6	1	6291.551	0.000
10	5	5	0	9	5	4	1	6297.215	-0.003
10	4	6	0	9	4	5	1	6368.124	0.000
10	2	8	0	9	2	7	1	6368.352	-0.052
11	1	11	0	10	1	10	1	6426.029	-0.003
11	0	11	0	10	0	10	1	6426.755	-0.009
10	1	9	1	9	1	8	0	6448.651	0.000
10	3	7	0	9	3	6	1	6466.547	0.009
10	3	8	1	9	3	7	0	6582.379	0.008
10	5	6	1	9	5	5	0	6646.305	0.000
10	4	7	1	9	4	6	0	6648.518	0.000
10	4	6	1	9	4	5	0	6725.216	0.003
11	1	11	1	10	1	10	0	6783.797	-0.012
11	0	11	1	10	0	10	0	6784.527	-0.014
4	1	4	0	3	1	3	1	2267.896	0.027
4	0	4	0	3	0	3	1	2313.620	0.050
4	2	3	0	3	2	2	1	2376.249	0.011
4	2	2	0	3	2	1	1	2444.746	-0.002
4	1	3	0	3	1	2	1	2465.131	-0.002

4	1	4	1	3	1	3	0	2625.803	0.005
4	0	4	1	3	0	3	0	2671.519	-0.002
4	2	3	1	3	2	2	0	2733.950	0.005
4	3	2	1	3	3	1	0	2753.572	0.014
4	3	1	1	3	3	0	0	2758.048	0.014
4	2	2	1	3	2	1	0	2802.497	-0.012
4	1	3	1	3	1	2	0	2822.854	-0.014
5	1	5	0	4	1	4	1	2869.447	0.011
5	0	5	0	4	0	4	1	2903.229	-0.005
5	2	4	0	4	2	3	1	3005.459	0.000
5	3	3	0	4	3	2	1	3042.280	-0.008
5	3	2	0	4	3	1	1	3057.411	0.005
5	1	4	0	4	1	3	1	3106.477	-0.006
5	2	3	0	4	2	2	1	3124.149	-0.012
5	1	5	1	4	1	4	0	3227.309	0.008
5	0	5	1	4	0	4	0	3261.120	-0.001
5	2	4	1	4	2	3	0	3363.033	0.005
5	3	3	1	4	3	2	0	3399.979	0.003
5	3	2	1	4	3	1	0	3415.105	0.000
5	1	4	1	4	1	3	0	3464.097	-0.008
6	1	6	0	5	1	5	1	3467.022	0.002
5	2	3	1	4	2	2	0	3481.857	0.001
6	0	6	0	5	0	5	1	3488.356	0.006
6	4	3	0	5	4	2	1	3688.613	-0.001
6	4	2	0	5	4	1	1	3690.742	-0.012
6	3	3	0	5	3	2	1	3725.583	-0.006
6	1	5	0	5	1	4	1	3731.903	-0.003
6	2	4	0	5	2	3	1	3800.862	-0.004
6	1	6	1	5	1	5	0	3824.860	0.007
6	0	6	1	5	0	5	0	3846.205	0.005
6	2	5	1	5	2	4	0	3986.082	0.006
6	3	4	1	5	3	3	0	4045.458	0.002
6	4	3	1	5	4	2	0	4046.263	0.002
6	4	2	1	5	4	1	0	4048.396	-0.007
10	3	7	1	9	3	6	0	6823.694	-0.005
11	3	9	0	10	3	8	1	6843.721	0.001
11	4	8	0	10	4	7	1	6936.597	0.011
11	2	9	0	10	2	8	1	6962.559	0.004
11	2	10	1	10	2	9	0	7011.831	0.003
12	1	12	0	11	1	11	1	7016.006	-0.008
11	1	10	1	10	1	9	0	7030.550	0.000
11	4	7	0	10	4	6	1	7064.843	0.019
11	3	8	0	10	3	7	1	7131.683	-0.001
11	3	9	1	10	3	8	0	7199.417	0.004
12	2	11	0	11	2	10	1	7247.210	-0.001
11	4	8	1	10	4	7	0	7293.326	0.010
11	5	7	1	10	5	6	0	7302.116	0.008
11	2	9	1	10	2	8	0	7318.424	-0.001
11	5	6	1	10	5	5	0	7320.911	0.011
12	0	12	1	11	0	11	0	7374.095	-0.024
11	4	7	1	10	4	6	0	7421.789	0.008
11	3	8	1	10	3	7	0	7488.677	-0.001
12	2	10	0	11	2	9	1	7543.448	0.019
12	2	11	1	11	2	10	0	7604.872	0.000
12	1	11	1	11	1	10	0	7615.115	0.003
12	3	10	1	11	3	9	0	7808.832	-0.005

12	2	10	1	11	2	9	0	7901.118	-0.014
6	2	5	2	5	2	4	3	3628.925	0.002
6	4	3	2	5	4	2	3	3688.785	-0.004
6	4	2	2	5	4	1	3	3690.920	-0.009
6	3	3	2	5	3	2	3	3725.761	-0.003
6	1	5	2	5	1	4	3	3732.086	0.002
6	2	4	2	5	2	3	3	3801.041	-0.001
6	1	6	3	5	1	5	2	3824.685	0.007
6	0	6	3	5	0	5	2	3846.032	0.008
6	2	5	3	5	2	4	2	3985.900	0.000
6	3	4	3	5	3	3	2	4045.281	0.002
6	4	3	3	5	4	2	2	4046.087	0.004
6	4	2	3	5	4	1	2	4048.224	-0.001
7	1	7	2	6	1	6	3	4061.750	0.004
7	0	7	2	6	0	6	3	4073.818	0.002
6	3	3	3	5	3	2	2	4083.008	-0.004
6	1	5	3	5	1	4	2	4089.131	-0.002
6	2	4	3	5	2	3	2	4158.295	-0.006
7	2	6	2	6	2	5	3	4246.557	0.000
7	3	5	2	6	3	4	3	4330.852	-0.009
7	1	6	2	6	1	5	3	4339.047	0.003
7	4	4	2	6	4	3	3	4339.485	0.000
7	4	3	2	6	4	2	3	4346.392	-0.004
7	1	7	3	6	1	6	2	4419.219	0.006
7	0	7	3	6	0	6	2	4431.298	0.004
7	2	5	2	6	2	4	3	4467.904	-0.002
7	2	6	3	6	2	5	2	4602.337	0.000
8	1	8	2	7	1	7	3	4654.249	0.001
8	0	8	2	7	0	7	3	4660.606	0.001
7	3	5	3	6	3	4	2	4687.920	0.001
7	5	3	3	6	5	2	2	4688.357	-0.001
7	1	6	3	6	1	5	2	4694.905	0.000
7	4	4	3	6	4	3	2	4696.643	0.004
7	4	3	3	6	4	2	2	4703.561	0.002
7	3	4	3	6	3	3	2	4763.623	0.000
7	2	5	3	6	2	4	2	4825.053	-0.006
8	2	7	2	7	2	6	3	4855.005	0.004
8	1	7	2	7	1	6	3	4930.740	0.000
8	3	6	2	7	3	5	3	4968.995	0.000
8	5	4	2	7	5	3	3	4981.772	-0.005
8	5	3	2	7	5	2	3	4982.716	-0.005
8	4	5	2	7	4	4	3	4991.202	-0.003
8	4	4	2	7	4	3	3	5009.252	-0.005
8	1	8	3	7	1	7	2	5011.712	0.007
8	0	8	3	7	0	7	2	5018.076	0.007
8	3	5	2	7	3	4	3	5096.114	0.002
8	2	6	2	7	2	5	3	5120.360	-0.004
9	1	9	2	8	1	8	3	5245.496	0.000
9	0	9	2	8	0	8	3	5248.684	-0.001
8	1	7	3	7	1	6	2	5287.185	0.001
8	3	6	3	7	3	5	2	5325.872	0.007
8	6	3	3	7	6	2	2	5330.419	0.018
8	6	2	3	7	6	1	2	5330.419	-0.004
8	5	4	3	7	5	3	2	5338.866	0.001
8	5	3	3	7	5	2	2	5339.807	-0.002
8	4	4	3	7	4	3	2	5366.275	0.000

8	3	5	3	7	3	4	2	5453.152	-0.002
9	2	8	2	8	2	7	3	5459.389	0.004
8	2	6	3	7	2	5	2	5477.368	-0.005
9	1	8	2	8	1	7	3	5510.792	0.003
9	3	7	2	8	3	6	3	5600.989	0.005
9	1	9	3	8	1	8	2	5602.950	0.004
9	0	9	3	8	0	8	2	5606.141	0.001
9	5	5	2	8	5	4	3	5634.627	-0.005
9	5	4	2	8	5	3	3	5637.600	-0.008
9	4	6	2	8	4	5	3	5642.558	0.000
9	4	5	2	8	4	4	3	5682.521	0.000
9	2	7	2	8	2	6	3	5754.773	0.000
9	3	6	2	8	3	5	3	5785.960	0.001
9	2	8	3	8	2	7	2	5817.140	0.002
10	0	10	2	9	0	9	3	5837.579	-0.004
9	1	8	3	8	1	7	2	5868.604	-0.001
9	6	4	3	8	6	3	2	5980.025	-0.001
9	6	3	3	8	6	2	2	5980.139	0.004
9	5	5	3	8	5	4	2	5991.538	-0.001
9	5	4	3	8	5	3	2	5994.519	-0.001
9	4	6	3	8	4	5	2	5999.373	0.000
9	4	5	3	8	4	4	2	6039.395	0.003
10	2	9	2	9	2	8	3	6058.700	0.003
10	1	9	2	9	1	8	3	6090.984	0.000
9	2	7	3	8	2	6	2	6111.567	-0.006
9	3	6	3	8	3	5	2	6142.892	0.000
10	1	10	3	9	1	9	2	6193.481	0.002
10	0	10	3	9	0	9	2	6195.024	-0.003
10	3	8	2	9	3	7	3	6225.921	0.000
10	5	6	2	9	5	5	3	6289.409	-0.004
10	4	7	2	9	4	6	3	6291.729	0.000
10	5	5	2	9	5	4	3	6297.395	0.000
10	2	8	2	9	2	7	3	6368.587	0.003
11	1	11	2	10	1	10	3	6426.199	-0.004
11	0	11	2	10	0	10	3	6426.919	-0.016
10	1	9	3	9	1	8	2	6448.474	0.000
10	3	7	2	9	3	6	3	6466.723	0.006
10	3	8	3	9	3	7	2	6582.197	0.005
10	5	6	3	9	5	5	2	6646.129	0.003
10	4	7	3	9	4	6	2	6648.340	0.003
11	2	10	2	10	2	9	3	6654.290	-0.008
10	5	5	3	9	5	4	2	6654.081	-0.038
11	1	10	2	10	1	9	3	6672.993	0.000
10	4	6	3	9	4	5	2	6725.037	0.005
10	2	8	3	9	2	7	2	6725.037	-0.002
11	1	11	3	10	1	10	2	6783.635	-0.003
11	0	11	3	10	0	10	2	6784.358	-0.012
10	3	7	3	9	3	6	2	6823.518	0.000
11	3	9	2	10	3	8	3	6843.902	0.006
11	4	8	2	10	4	7	3	6936.769	0.006
11	2	9	2	10	2	8	3	6962.737	0.004
11	2	10	3	10	2	9	2	7011.656	0.003
11	1	10	3	10	1	9	2	7030.377	0.001
11	4	7	2	10	4	6	3	7065.012	0.010
11	3	8	2	10	3	7	3	7131.871	0.006
11	3	9	3	10	3	8	2	7199.242	0.008

12	2	11	2	11	2	10	3	7247.385	0.001
12	1	11	2	11	1	10	3	7257.599	-0.005
11	4	8	3	10	4	7	2	7293.144	0.009
11	5	7	3	10	5	6	2	7301.937	0.010
11	2	9	3	10	2	8	2	7318.247	0.000
11	5	6	3	10	5	5	2	7320.725	0.006
12	1	12	3	11	1	11	2	7373.593	-0.015
12	0	12	3	11	0	11	2	7373.934	-0.014
11	4	7	3	10	4	6	2	7421.610	0.011
11	3	8	3	10	3	7	2	7488.497	0.002
12	2	10	2	11	2	9	3	7543.611	0.005
12	2	11	3	11	2	10	2	7604.706	0.007
12	1	11	3	11	1	10	2	7614.935	-0.001
12	3	10	3	11	3	9	2	7808.664	0.004
6	0	6	2	5	0	5	3	3488.527	0.004
5	2	3	3	4	2	2	2	3481.679	0.000
6	1	6	2	5	1	5	3	3467.204	0.010
5	1	4	3	4	1	3	2	3463.923	-0.005
5	3	2	3	4	3	1	2	3414.926	-0.003
5	3	3	3	4	3	2	2	3399.801	0.002
5	4	1	3	4	4	0	2	3397.825	-0.003
5	2	4	3	4	2	3	2	3362.857	0.005
5	0	5	3	4	0	4	2	3260.949	0.002
5	1	5	3	4	1	4	2	3227.134	0.007
5	2	3	2	4	2	2	3	3124.337	0.000
5	1	4	2	4	1	3	3	3106.658	-0.001
5	3	2	2	4	3	1	3	3057.578	-0.003
5	3	3	2	4	3	2	3	3042.466	0.002
5	2	4	2	4	2	3	3	3005.634	0.000
5	0	5	2	4	0	4	3	2903.415	0.006
5	1	5	2	4	1	4	3	2869.617	0.005
4	1	3	3	3	1	2	2	2822.682	-0.009
4	2	2	3	3	2	1	2	2802.320	-0.012
4	3	1	3	3	3	0	2	2757.840	-0.017
4	3	2	3	3	3	1	2	2753.353	-0.027
4	2	3	3	3	2	2	2	2733.780	0.012
4	0	4	3	3	0	3	2	2671.342	-0.004
4	1	4	3	3	1	3	2	2625.622	0.000
4	1	3	2	3	1	2	3	2465.308	-0.001
4	2	2	2	3	2	1	3	2444.918	-0.006
4	2	3	2	3	2	2	3	2376.405	-0.006
4	1	4	2	3	1	3	3	2268.043	0.000
6	2	5	5	5	2	4	4	3986.616	0.007
7	1	7	4	6	1	6	5	4061.048	0.010
7	0	7	4	6	0	6	5	4073.111	0.003
6	1	5	5	5	1	4	4	4089.836	-0.004
6	2	4	5	5	2	3	4	4159.004	-0.003
7	2	6	4	6	2	5	5	4245.876	-0.002
7	4	3	4	6	4	2	5	4345.686	-0.004
7	1	7	5	6	1	6	4	4419.942	0.020
7	0	7	5	6	0	6	4	4432.008	0.005
7	2	6	5	6	2	5	4	4603.051	0.005
8	0	8	4	7	0	7	5	4659.894	-0.001
7	4	3	5	6	4	2	4	4704.271	0.004
7	3	4	5	6	3	3	4	4764.331	0.000
7	2	5	5	6	2	4	4	4825.760	-0.006

8	2	7	4	7	2	6	5	4854.307	0.005
8	1	7	4	7	1	6	5	4930.061	0.000
8	4	5	4	7	4	4	5	4990.494	-0.004
8	4	4	4	7	4	3	5	5008.553	0.001
6	3	4	5	5	3	3	4	4045.967	-0.018
6	4	3	5	5	4	2	4	4046.790	0.000
6	3	3	5	5	3	2	4	4083.725	0.005
7	3	5	4	6	3	4	5	4330.138	-0.016
7	1	6	4	6	1	5	5	4338.343	0.006
7	3	4	4	6	3	3	5	4405.775	0.001
8	1	8	4	7	1	7	5	4653.546	0.007
7	5	3	5	6	5	2	4	4689.060	-0.005
7	5	2	5	6	5	1	4	4689.302	-0.005
7	1	6	5	6	1	5	4	4695.576	-0.008
7	4	4	5	6	4	3	4	4697.356	0.008
8	3	6	4	7	3	5	5	4968.291	0.003
8	5	4	4	7	5	3	5	4981.064	-0.007
8	5	3	4	7	5	2	5	4981.998	-0.016
8	1	8	5	7	1	7	4	5012.415	0.000
8	0	8	5	7	0	7	4	5018.783	0.004
8	3	5	4	7	3	4	5	5095.404	-0.003
8	2	6	4	7	2	5	5	5119.650	-0.009
8	2	7	5	7	2	6	4	5212.052	0.005
9	1	9	4	8	1	8	5	5244.795	0.009
9	0	9	4	8	0	8	5	5247.977	0.001
8	1	7	5	7	1	6	4	5287.887	0.005
8	6	3	5	7	6	2	4	5331.125	0.016
8	6	2	5	7	6	1	4	5331.125	-0.006
8	5	4	5	7	5	3	4	5339.577	0.004
8	5	3	5	7	5	2	4	5340.529	0.010
8	4	5	5	7	4	4	4	5348.907	0.000
8	4	4	5	7	4	3	4	5366.992	0.008
8	3	5	5	7	3	4	4	5453.854	-0.006
9	2	8	4	8	2	7	5	5458.685	0.006
8	2	6	5	7	2	5	4	5478.082	0.001
9	1	8	4	8	1	7	5	5510.089	-0.002
9	3	7	4	8	3	6	5	5600.280	0.004
9	1	9	5	8	1	8	4	5603.659	0.002
9	0	9	5	8	0	8	4	5606.848	-0.003
9	5	5	4	8	5	4	5	5633.922	-0.003
9	5	4	4	8	5	3	5	5636.904	0.002
9	4	6	4	8	4	5	5	5641.855	0.002
9	4	5	4	8	4	4	5	5681.812	-0.003
9	2	7	4	8	2	6	5	5754.073	0.005
9	3	6	4	8	3	5	5	5785.264	0.009
10	1	10	4	9	1	9	5	5835.321	-0.003
10	0	10	4	9	0	9	5	5836.876	0.004
9	3	7	5	8	3	6	4	5958.326	0.003
9	6	4	5	8	6	3	4	5980.725	-0.010
9	6	3	5	8	6	2	4	5980.847	0.003
9	5	5	5	8	5	4	4	5992.242	-0.006
9	5	4	5	8	5	3	4	5995.224	-0.005
9	4	6	5	8	4	5	4	6000.088	0.007
9	4	5	5	8	4	4	4	6040.097	-0.002
10	1	9	4	9	1	8	5	6090.278	-0.001
9	2	7	5	8	2	6	4	6112.278	-0.002

9	3	6	5	8	3	5	4	6143.596	-0.001
10	1	10	5	9	1	9	4	6194.188	-0.003
10	0	10	5	9	0	9	4	6195.729	-0.011
10	3	8	4	9	3	7	5	6225.213	0.002
10	5	6	4	9	5	5	5	6288.708	0.001
10	5	5	4	9	5	4	5	6296.686	-0.002
10	4	6	4	9	4	5	5	6367.600	0.002
10	2	8	4	9	2	7	5	6367.881	0.004
11	1	11	4	10	1	10	5	6425.486	-0.005
10	1	9	5	9	1	8	4	6449.183	0.000
10	3	7	4	9	3	6	5	6466.012	-0.001
10	3	8	5	9	3	7	4	6582.911	0.007
10	4	7	5	9	4	6	4	6649.061	0.014
11	2	10	4	10	2	9	5	6653.596	0.005
10	5	5	5	9	5	4	4	6654.834	0.004
11	1	10	4	10	1	9	5	6672.284	-0.001
10	4	6	5	9	4	5	4	6725.746	0.007
10	2	8	5	9	2	7	4	6725.746	-0.001
11	0	11	5	10	0	10	4	6785.064	-0.020
10	3	7	5	9	3	6	4	6824.232	0.008
11	3	9	4	10	3	8	5	6843.201	0.012
11	4	8	4	10	4	7	5	6936.059	0.004
11	2	9	4	10	2	8	5	6962.028	0.002
11	2	10	5	10	2	9	4	7012.367	0.005
12	1	12	4	11	1	11	5	7015.461	-0.007
12	0	12	4	11	0	11	5	7015.785	-0.023
11	1	10	5	10	1	9	4	7031.079	-0.007
11	4	7	4	10	4	6	5	7064.302	0.003
11	3	8	4	10	3	7	5	7131.163	0.001
11	3	9	5	10	3	8	4	7199.952	0.005
12	1	11	4	11	1	10	5	7256.893	-0.001
11	4	8	5	10	4	7	4	7293.854	0.008
11	2	9	5	10	2	8	4	7318.942	-0.009
11	5	6	5	10	5	5	4	7321.432	0.003
12	0	12	5	11	0	11	4	7374.625	-0.039
11	4	7	5	10	4	6	4	7422.315	0.009
11	3	8	5	10	3	7	4	7489.201	0.000
12	2	10	4	11	2	9	5	7542.908	0.007
12	1	11	5	11	1	10	4	7615.649	0.000
12	2	10	5	11	2	9	4	7901.629	0.002
13	1	13	5	12	1	12	4	7964.243	0.035
12	1	12	5	11	1	11	4	7374.333	0.008
9	2	8	5	8	2	7	4	5817.787	-0.050
6	0	6	5	5	0	5	4	3846.737	0.005
6	1	6	5	5	1	5	4	3825.395	0.009
6	2	4	4	5	2	3	5	3800.340	0.002
6	1	5	4	5	1	4	5	3731.376	0.000
6	3	3	4	5	3	2	5	3725.047	-0.012
6	4	2	4	5	4	1	5	3690.215	-0.008
6	3	4	4	5	3	3	5	3687.357	-0.005
6	2	5	4	5	2	4	5	3628.209	-0.005
6	0	6	4	5	0	5	5	3487.829	0.013
5	2	3	5	4	2	2	4	3482.388	0.002
6	1	6	4	5	1	5	5	3466.498	0.011
5	3	2	5	4	3	1	4	3415.628	-0.007
5	3	3	5	4	3	2	4	3400.467	-0.038

5	2	4	5	4	2	3	4	3363.583	0.024
5	0	5	5	4	0	4	4	3261.664	0.010
5	1	5	5	4	1	4	4	3227.840	0.006
5	2	3	4	4	2	2	5	3123.621	-0.011
5	1	4	4	4	1	3	5	3105.936	-0.017
5	3	3	4	4	3	2	5	3041.747	-0.010
5	2	4	4	4	2	3	5	3004.926	-0.001
5	0	5	4	4	0	4	5	2902.714	0.011
5	1	5	4	4	1	4	5	2868.912	0.007
4	1	3	5	3	1	2	4	2823.382	-0.015
4	3	2	5	3	3	1	4	2754.063	-0.024
4	0	4	5	3	0	3	4	2672.057	0.004
4	1	4	5	3	1	3	4	2626.334	0.005

Table S2. Theoretical cartesian coordinates (Å) for conformer 1.

	B3LYP-D3BJ/def2-TZPV		
Atom type	*a*	*b*	*c*
C	-1.190	0.218	-1.768
C	-1.223	-0.389	-0.513
C	0.015	-0.676	0.093
C	1.249	-0.372	-0.514
C	1.204	0.234	-1.767
C	0.003	0.529	-2.392
H	-2.112	0.459	-2.272
H	2.123	0.487	-2.270
H	0.000	1.002	-3.365
C	-2.568	-0.714	0.164
C	-2.718	0.066	1.489
H	-3.679	-0.166	1.952
H	-1.943	-0.139	2.228
H	-2.685	1.138	1.294
C	-3.762	-0.298	-0.708
H	-3.767	-0.822	-1.664
H	-4.688	-0.547	-0.188
H	-3.768	0.774	-0.902
C	-2.718	-2.235	0.394
H	-2.672	-2.761	-0.560
H	-1.951	-2.678	1.030
H	-3.684	-2.448	0.856
C	2.590	-0.684	0.168
C	2.704	0.085	1.499
H	1.919	-0.193	2.197
H	3.669	-0.125	1.966
H	2.643	1.160	1.323
C	3.783	-0.257	-0.700
H	4.708	-0.499	-0.175
H	3.800	-0.781	-1.657
H	3.784	0.816	-0.893
C	2.729	-2.199	0.413
H	2.676	-2.743	-0.532
H	3.699	-2.412	0.869
H	1.951	-2.577	1.070
O	0.081	-1.267	1.330
H	-0.804	-1.438	1.657
Ar	-0.042	2.883	0.661

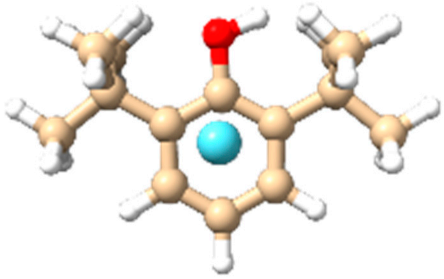

Table S3. Theoretical cartesian coordinates (Å) for conformer 2.

	B3LYP-D3BJ/def2-TZPV		
Atom type	*a*	*b*	*c*
C	-0.882	2.619	0.000
C	0.021	1.556	-0.000
C	-0.518	0.254	-0.000
C	-1.905	0.006	0.000
C	-2.746	1.116	0.000
C	-2.249	2.409	0.000
H	-0.516	3.633	0.000
H	-3.816	0.974	0.000
H	-2.927	3.252	0.000
C	1.539	1.817	-0.000
C	2.196	1.243	-1.275
H	3.270	1.438	-1.262
H	2.063	0.170	-1.411
H	1.772	1.724	-2.158
C	1.860	3.320	0.000
H	1.465	3.819	0.885
H	2.943	3.454	0.000
H	1.464	3.819	-0.884
C	2.196	1.242	1.275
H	1.772	1.723	2.158
H	2.064	0.169	1.411
H	3.270	1.438	1.262
C	-2.475	-1.422	-0.000
C	-2.023	-2.179	-1.265
H	-0.943	-2.275	-1.315
H	-2.459	-3.180	-1.270
H	-2.366	-1.658	-2.161
C	-4.010	-1.420	0.000
H	-4.365	-2.452	0.000
H	-4.419	-0.931	0.885
H	-4.419	-0.931	-0.885
C	-2.023	-2.179	1.264
H	-2.366	-1.658	2.161
H	-2.459	-3.181	1.270
H	-0.943	-2.275	1.315
O	0.291	-0.852	-0.000
H	1.212	-0.585	-0.000
Ar	3.462	-2.340	0.000

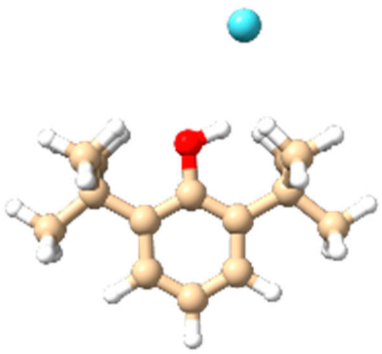

Table S4. Theoretical cartesian coordinates (Å) for conformer 3.

	B3LYP-D3BJ/def2-TZPV		
Atom type	*a*	*b*	*c*
C	1.013	0.646	0.000
C	0.323	-0.565	0.000
C	-1.084	-0.502	-0.000
C	-1.791	0.716	0.000
C	-1.035	1.885	0.000
C	0.349	1.860	0.000
H	2.090	0.650	0.000
H	-1.533	2.842	0.000
H	0.910	2.785	0.000
C	1.090	-1.902	0.000
C	0.776	-2.717	-1.274
H	1.342	-3.651	-1.267
H	-0.274	-2.977	-1.400
H	1.065	-2.149	-2.159
C	2.611	-1.685	0.000
H	2.943	-1.141	0.884
H	3.110	-2.655	0.000
H	2.943	-1.141	-0.884
C	0.776	-2.717	1.274
H	1.065	-2.149	2.159
H	-0.275	-2.977	1.400
H	1.342	-3.651	1.267
C	-3.328	0.764	-0.000
C	-3.885	0.081	-1.265
H	-3.606	-0.966	-1.316
H	-4.976	0.147	-1.270
H	-3.514	0.582	-2.161
C	-3.851	2.208	0.000
H	-4.942	2.189	-0.000
H	-3.531	2.759	0.885
H	-3.531	2.759	-0.885
C	-3.886	0.081	1.265
H	-3.514	0.582	2.161
H	-4.976	0.147	1.270
H	-3.606	-0.966	1.316
O	-1.847	-1.643	-0.000
H	-1.278	-2.415	-0.000
Ar	4.893	1.499	-0.000

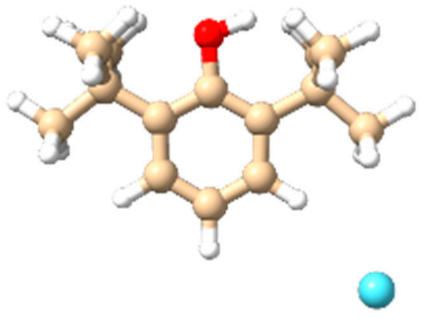

Table S5. Theoretical cartesian coordinates (Å) for conformer 4.

	B3LYP-D3BJ/def2-TZPV		
Atom type	*a*	*b*	*c*
C	-0.432	2.206	-0.000
C	-0.034	0.869	-0.000
C	-1.055	-0.103	-0.000
C	-2.424	0.229	0.000
C	-2.745	1.584	0.000
C	-1.767	2.566	-0.000
H	0.311	2.986	-0.000
H	-3.780	1.887	0.000
H	-2.047	3.611	-0.000
C	1.460	0.495	0.000
C	1.830	-0.295	-1.274
H	2.895	-0.534	-1.269
H	1.289	-1.234	-1.400
H	1.619	0.306	-2.159
C	2.359	1.741	0.000
H	2.197	2.358	0.884
H	3.403	1.428	0.000
H	2.197	2.358	-0.884
C	1.830	-0.295	1.274
H	1.620	0.306	2.159
H	1.289	-1.234	1.400
H	2.895	-0.534	1.269
C	-3.522	-0.846	0.000
C	-3.416	-1.721	-1.265
H	-2.467	-2.246	-1.316
H	-4.220	-2.461	-1.270
H	-3.519	-1.105	-2.161
C	-4.926	-0.223	0.000
H	-5.668	-1.024	0.000
H	-5.103	0.389	0.885
H	-5.103	0.389	-0.885
C	-3.416	-1.721	1.265
H	-3.519	-1.106	2.161
H	-4.219	-2.461	1.270
H	-2.466	-2.246	1.316
O	-0.759	-1.443	-0.000
H	0.191	-1.567	-0.000
Ar	5.747	-0.725	0.000

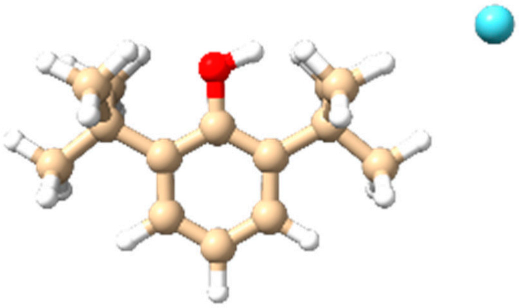

Table S6. Theoretical cartesian coordinates (Å) for conformer 5.

	B3LYP-D3BJ/def2-TZPV		
Atom type	*a*	*b*	*c*
C	2.194	1.818	0.123
C	2.230	0.424	0.144
C	1.048	-0.253	-0.214
C	-0.133	0.419	-0.584
C	-0.094	1.811	-0.582
C	1.050	2.509	-0.234
H	3.074	2.382	0.390
H	-0.975	2.370	-0.858
H	1.050	3.591	-0.241
C	3.518	-0.320	0.545
C	4.039	-1.189	-0.621
H	4.960	-1.695	-0.326
H	3.343	-1.956	-0.962
H	4.256	-0.559	-1.484
C	4.657	0.654	0.887
H	4.401	1.297	1.730
H	5.544	0.083	1.163
H	4.919	1.285	0.038
C	3.291	-1.170	1.815
H	2.986	-0.528	2.642
H	2.523	-1.938	1.717
H	4.218	-1.674	2.097
C	-1.413	-0.338	-0.972
C	-1.158	-1.221	-2.210
H	-0.398	-1.974	-2.022
H	-2.082	-1.729	-2.497
H	-0.835	-0.608	-3.054
C	-2.554	0.624	-1.334
H	-3.437	0.042	-1.600
H	-2.825	1.270	-0.498
H	-2.302	1.252	-2.190
C	-1.904	-1.200	0.206
H	-2.112	-0.572	1.075
H	-2.829	-1.710	-0.068
H	-1.171	-1.949	0.492
O	0.989	-1.624	-0.222
H	1.834	-1.988	0.047
Ar	-5.440	0.012	1.110

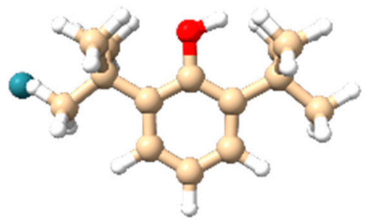

Supplement: Supplementary file 1 [file molecules-28-08111-s001.zip › molecules-2715729-supplementary.pdf]
